# Pharmacoinformatics-based identification of transmembrane protease serine-2 inhibitors from *Morus Alba* as SARS-CoV-2 cell entry inhibitors

**DOI:** 10.1007/s11030-021-10209-3

**Published:** 2021-03-30

**Authors:** Anshul Shakya, Rupesh V. Chikhale, Hans Raj Bhat, Fatmah Ali Alasmary, Tahani Mazyad Almutairi, Surajit Kumar Ghosh, Hassna Mohammed Alhajri, Siham A. Alissa, Shuchi Nagar, Md Ataul Islam

**Affiliations:** 1 Department of Pharmaceutical Sciences, Faculty of Science and Engineering, Dibrugarh University, Dibrugarh, Assam 786 004 India; 2 School of Pharmacy, University of East Anglia, Norwich Research Park, Norwich, NR5 7TJ UK; 3 Chemistry Department, College of Science, King Saud University, P.O. Box 2455, Riyadh, 11451 Saudi Arabia; 4 Department of Chemistry, College of Science, Princess Nourah bint Abdulrahman University, Riyadh, 11671 Saudi Arabia; 5 Bioinformatics Research Centre, Dr. D. Y. Patil Biotechnology & Bioinformatics Institute, Dr. D. Y. Patil Vidyapeeth, Tathawade, Pune, India; 6 Division of Pharmacy and Optometry, School of Health Sciences, Faculty of Biology, Medicine and Health, University of Manchester, Manchester, UK; 7 School of Health Sciences, University of Kwazulu-Natal, Westville Campus, Durban, South Africa; 8 Department of Chemical Pathology, Faculty of Health Sciences, University of Pretoria, Pretoria, South Africa

**Keywords:** *Morus alba* Linn., SARS-CoV-2, TMPRSS2, Virtual screening, Molecular docking

## Abstract

**Abstract:**

Transmembrane protease serine-2 (TMPRSS2) is a cell-surface protein expressed by epithelial cells of specific tissues including those in the aerodigestive tract. It helps the entry of novel coronavirus (n-CoV) or Severe Acute Respiratory Syndrome Coronavirus 2 (SARS-CoV-2) in the host cell. Successful inhibition of the TMPRSS2 can be one of the crucial strategies to stop the SARS-CoV-2 infection. In the present study, a set of bioactive molecules from *Morus alba* Linn. were screened against the TMPRSS2 through two widely used molecular docking engines such as Autodock vina and Glide. Molecules having a higher binding affinity toward the TMPRSS2 compared to Camostat and Ambroxol were considered for *in-silico* pharmacokinetic analyses. Based on acceptable pharmacokinetic parameters and drug-likeness, finally, five molecules were found to be important for the TMPRSS2 inhibition. A number of bonding interactions in terms of hydrogen bond and hydrophobic interactions were observed between the proposed molecules and ligand-interacting amino acids of the TMPRSS2. The dynamic behavior and stability of best-docked complex between TRMPRSS2 and proposed molecules were assessed through molecular dynamics (MD) simulation. Several parameters from MD simulation have suggested the stability between the protein and ligands. Binding free energy of each molecule calculated through MM-GBSA approach from the MD simulation trajectory suggested strong affection toward the TMPRSS2. Hence, proposed molecules might be crucial chemical components for the TMPRSS2 inhibition.

**Graphic abstract:**

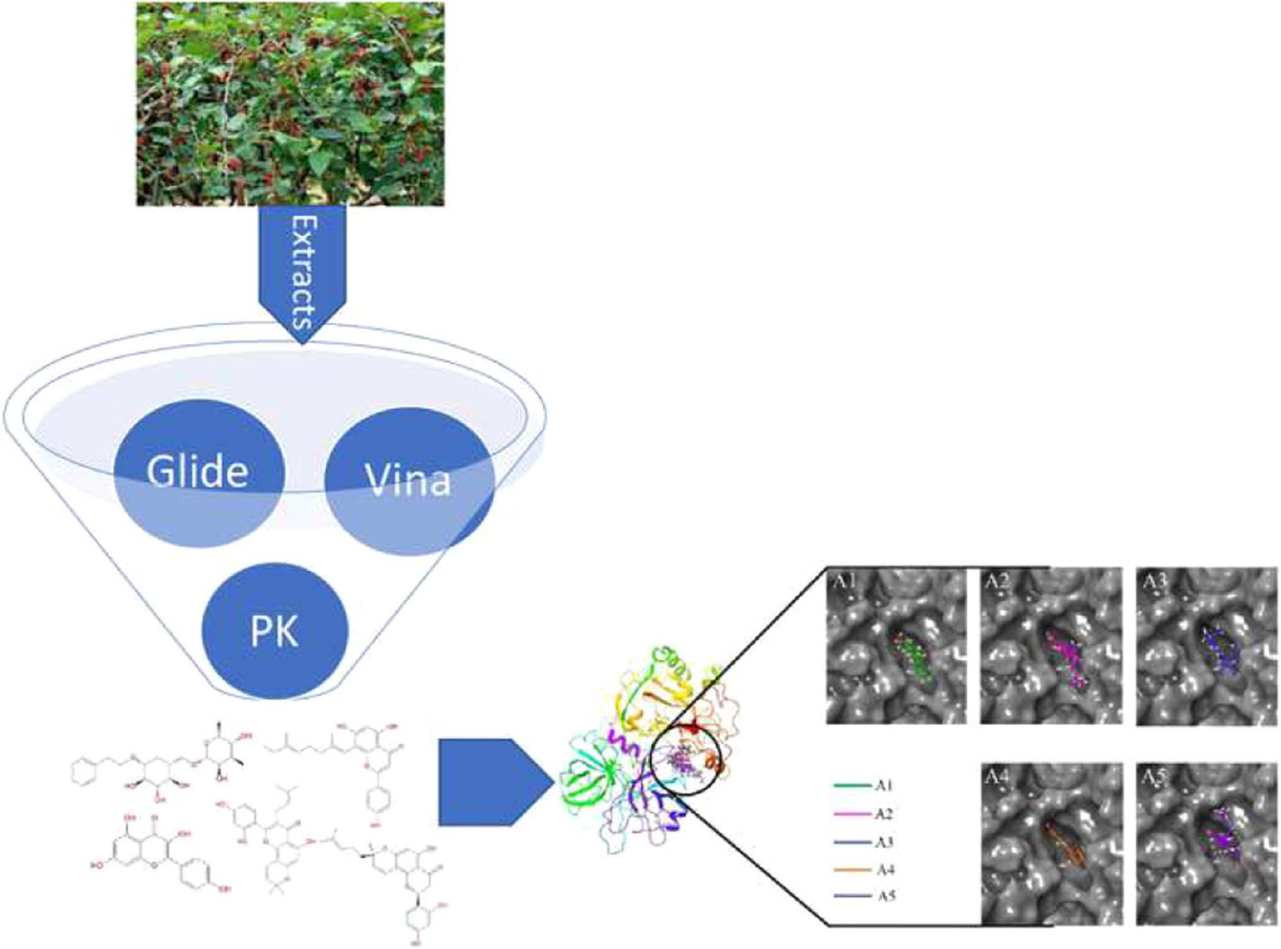

**Supplementary Information:**

The online version contains supplementary material available at 10.1007/s11030-021-10209-3.

## Introduction

The pandemic outbreak of the novel Coronavirus (n-CoV) or Severe Acute Respiratory Syndrome Coronavirus 2 (SARS-CoV-2) causes the respiratory illness and named as coronavirus disease-2019 (COVID-19) worldwide [1]. So far, this deadly disease left millions of human being infected and thousands of deaths [2]. Of these unfortunate deaths, United States of America shares about 55%, Europe contributes almost 25% followed by South-East Asia about 10% [3]. Notably, with time progress the number of infected individuals and figures related to death are gradually raising. Thus, there is an urgent need for effective and preventive therapeutic intervention against COVID-19. A number of drug discovery approaches including molecular docking, molecular similarity, pharmacophore and artificial intelligence can be used to facilitate the drug discovery efforts for COVID-19 [4–6]. The availability of experimental drug targets associated with COVID-19 is the key for clinical/biologic evaluations of drug efficacies, investigations of therapeutic mechanisms and searches of drug-repurposing opportunities [7, 8].

Genomic studies suggest high sequence identity between the genome of existing SARS-CoV and current SARS-CoV-2 [9]. As the most critical step during infection, SARS-CoV-2 uses its Spike (S) protein receptor-binding domain (S-RBD) to engage with the host cell receptor angiotensin-converting enzyme 2 (ACE2) [10]. The SARS-CoV-2 needs to enter into the cells, which is allowed through ACE2 via the action of transmembrane protease serine-2 (TMPRSS2). The TMPRSS2 is a cell-surface protein that is expressed by epithelial cells of specific tissues including those in the aerodigestive tract [11]. The TMPRSS2 triggers the priming of the virus’s S protein by assisting the cleavage of the S proteins at the S1/S2 and S2 sites [12]. Thus, the cleavage step or the TMPRSS2 activity is necessary for the virus-host cell membrane fusion and cell entry [13]. Apart from the said pathological role, ACE2 also possesses essential physiological roles such as regulation of vasoconstriction and blood pressure, which might become difficult to target ACE2 in therapies [14]. Interestingly, the TMPRSS2-expressing cells are more susceptible to SARS-CoV-2 infection and knockout mouse models show that lack of TMPRSS2 in the airways reduces the severity of lung pathology after SARS-CoV and MERS-CoV infection [15]. Therefore, targeting the TMPRSS2 is a rational approach to manage the spread and infection caused by SARS-CoV-2 and to treat the COVID-19 patients [16, 17].

Medicinal plants have historically proven their value as a source of molecules with therapeutic potential, and nowadays still represent an important tool for the identification of novel drug leads. A range of secondary metabolites are the potential bioactive compounds, which were naturally selected for thousands of years to improve the specificity and cover a very wide range of functions, depending on the origin, the habitat and the specific activity carried out in the organism of origin [18, 19]. *Morus alba* Linn. (Family: Moraceae), named as ‘white mulberry’, is one of the deciduous medium-sized trees cultivated in the tropical countries for rearing silkworms and ruminants [20]. The natives of India use the leaves of *M. alba* to treat cough, asthma, bronchitis, eye infection, headache and dizziness [21]. The inhabitants of lesser Himalayas in Pakistan take fresh fruits and leaves decoction orally for throat ache [22]. The root bark has been used in traditional Korean medicine for upper respiratory diseases [23]. The European countries, *M. alba* is welcomed as a ‘superfood’ due to the presence of the high amount of bioactive constituents which are beneficial to promote health and longevity [24]. The *M. alba* juice and the seed have been reported to possess anti-viral activities against influenza viruses, A/Brisbane/59/2007 (H1N1) (BR59), pandemic A/Korea/01/2009(H1N1) (KR01), A/Brisbane/10/2007(H3N2) (BR10), and B/Florida/4/2006 (FL04) [25]. The aqueous extract of the *M. alba* exhibited potential anti-dengue activity against varied stages of the dengue virus replication cycle due to the presence of flavonoids [26]. A report suggests that *M. alba* juice and its fractions may inhibit internalization and replication of murine norovirus-1 (MNV-1), whereas it may influence adherence or internalization of feline calicivirus-F9 (FCV-F9) virions [27]. The *M. alba* extract has also been effective against *Herpes Simplex* Virus type 1 (HSV-1) in an *in-vitro* finding on the Vero cell lines, which might be due to available flavonoid compounds [28]. Moreover, phenolic compounds from *M. alba* root bark such as moralbanone, kuwanon S, mulberroside C, cyclomorusin, eudraflavone B hydroperoxide, oxydihydromorusin, leachianone G and α-acetyl-amyrin have promising anti-infective property specifically against the replication of HSV-1 or herpes simplex virus 2 (HSV-2) possibly via by inhibiting HSV-1 DNA polymerase and HSV-2 protease [29]. Additionally, mulberrofuran G and isomulberrofuran G isolated from the root bark of *M. alba* showed moderate activity by inhibiting hepatitis B virus (HBV) DNA replication in an anti-HBV assay on the HepG 2.2.15 cell line [30]. Iminosugar derivatives of 1-deoxynojirimycin have demonstrated anti-viral activity against bovine viral diarrhea virus (BVDV) and GB virus-B (GBV-B), both members of the Flaviviridae family, and against woodchuck hepatitis virus (WHV) and hepatitis B virus (HBV), both members of the Hepadnaviridae family of viruses [31]. Furthermore, a recent investigation suggests the efficacy of the water and water-alcohol plant extracts of the leaves and stem bark of *M. alba* against the viral respiratory infections caused by human coronavirus (HCoV 229E) and picornaviruses [32].

Therefore, in the view of the facts mentioned above, this study was aimed to investigate the TMPRSS2 inhibitory potential of bioactive isolated from the *M. alba* using *in-silico* modeling. Each of the bioactive compounds in this collection has been optimized for efficacy, safety, and bioavailability using high-throughput virtual screening tools. This enables the leveraging of considerable investments in research and development to compress the timeline required for drug discovery and development. Molecular docking is an essential and widely used pharmacoinformatics approach in which the favorable binding mode of the small molecules is predicted in the target site through conformational analyses. Due to its fast in execution, trustworthy and ease to use, molecular docking has become favorable to the wider community of researchers from academia and industry. Molecular docking is extremely successful to screen larger datasets of small molecular to achieve potential lead-like molecules for a specific target. *In-silico* pharmacokinetic and drug-likeness assessment are critical strategies to select lead-like molecules from a pool of initial hits [33]. Molecular dynamics (MD) simulation is an important method to assess the behavior of protein–ligand complex in dynamic states. Hence, the above approaches might be crucial to identify potential molecules for a certain target.

## Materials and methods

A dataset of small molecules belongs to *M. alba* Linn. was screened against the TMPRSS2 through two separate molecular docking engines, Glide [34] of Schrodinger suite and Autodock vina (ADV) [35]. By following a number of screening criteria, finally, five molecules were found to be potential for the TMPRSS2 inhibition. The all-atoms MD simulation [36] was carried out to explore the stability of complexes between the TMPRSS2 and the final proposed molecules. The MD simulation trajectory was also used to study the affinity of the final molecules toward the TMPRSS2 through the molecular mechanics-generalized born surface area (MM-GBSA) [37] approach.

### Small molecular dataset and protein structure preparation

In order to screen the potential bioactive compounds for the effective inhibition of the TMPRSS2, a set of 144 reported bioactive compounds of *M. alba* Linn. was retrieved from the PubChem [38]. The two-dimensional representation of each 144 molecules is given in Table S1 (Supplementary file). It is already been proved that the different parts of the *M. alba* possess strong anti-viral activity. Hence, screening the above molecules through already established druggable targets such as the TMPRSS2 can give few crucial molecules for successful inhibition the same. TMPRSS2 is one of the crucial targets to stop SARS-CoV-2 infection. Recently our group has developed the 3D coordinates of the TMPRSS2 through the homology modeling [39] followed by screening the Selleckchem database (https://www.selleckchem.com/) and it has been published [17]. The same TMPRSS structure was considered to screen the above 144 bioactive compounds belong to the *M. alba* Linn.

Before docking, the entire dataset of small molecules was prepared through the LigPrep [40] module of the Schrodinger suite. The maximum number of stereoisomer generation was allowed to 32 at a pH of 7.0 ± 2.0. The ‘Epik’ functionality of the LigPrep [40] was used to retain the chirality and ionization states of the molecules. The OPLS3 force field [41] was used for the optimization of the structure. Followed by successful validation of TMPRSS2 structure [17] generated through the homology model [39], the same was considered for MD simulation to minimized and remove the steric clashes. Details analysis and protocol can be found in our previous publication [17]. Final coordinates of TMPRSS2 after a 100 ns all-atoms MD simulation were considered for molecular docking study. The SiteMap [42] and MOE [43] tools were used to find the active site. Receptor site confining the His18, Gln21, Glu23, Asn24, Pro25, Val49, Pro50, Gln51, Tyr52, Ala53, Pro54, Arg55, Gln59, Val65, Gln68, Pro69, Val96, Gly97, Ala98, Ala99, Ala101, Met371, Met372, Leu373, Gln374, Glu376, Gln377, Leu378, Thr447, Lys449, Asn450, Asn451, Ile452, and Trp454 was considered as the active site. The Grid generation module was used to develop the grid around the above amino acids. It is important to note that the catalytic triads of TMPRSS2 were reported as His296, Asp345 and Ser441 [44]. The grid generated by confining the active site residues was also found to contains the catalytic triads.

To dock the entire set of molecules through ADV, the Autodock tools (ADT) [45] was used to prepare and convert all the molecules into.pdbqt format. Similarly, the ADT interface was used to prepare the TMPRSS2 for the input of the ADV program. In protein preparation, the hydrogen and Gasteiger charge were added and atom type was assigned as AD4 (Autodock 4) type. The prepared protein was saved as. pdbqt for the input of ADV. The grid coordinate was assigned as 48.554, 60.627 and 44.601 along the x-, y- and z-axis, respectively. To accommodate all the amino acids present in the active site, the grid size was set to 60 Å × 60 Å × 60 Å along the x-, y- and z-axis , respectively.

### Virtual screening

Virtual screening of phytochemicals through the pharmacoinformatics approach has become a vital tool in the drug discovery and development phase. In the current study, the published 3D structure of the TMPRSS2 [17] was used as a target for virtual screening of a set of 144 reported bioactive compounds of *M. alba*, which were retrieved from the PubChem [38]. The molecular docking simulation studies were performed on two of the most widely used and trusted steadfast docking simulation tools, i.e., ADV program [35] and Glide module [34] of the Schrodinger suite. Out of five standard drug molecules, Camostat and Ambroxolwere selected as control molecules based on binding energy and binding interactions. Details about the selection procedure can be found in our previous publication [17]. In Glide docking, molecules having a higher Glide score compare to Camostat and Ambroxol were removed for further assessment. On the parallel approach, entire molecular set along with Camostat and Ambroxol were docked in the TMPRSS2 through ADV. The binding free energy of Camostat and Ambroxol obtained from ADV was used as a threshold to remove the low potential molecules. Molecules found common in both Glide and ADV docking studies and screen out through the threshold value of Camostat and Ambroxol were further used to calculate the binding free energy through Prime-MMGBSA. Molecules having Prime-MMGBSA binding energy higher than Camostat and Ambroxol were removed. Finally, a number of drug-likeness and pharmacokinetic parameters included Lipinski’s rule of five (LoF), Gastro intestine (GI) absorption, total polar surface area (TPSA), blood–brain barrier (BBB) permeation, Veber’s rule and synthetic accessibility were used to remove the less potential molecules.

### Molecular dynamics simulation

The behavior and dynamic nature of the protein–ligand complex were assessed through MD simulation study. Also, this approach helps in binding interactions between protein and ligand along with energetically favorable conformation analyses. Complexes between the TMPRSS2 and final proposed inhibitors were considered for all-atoms 100 ns MD simulation. The Amber18 [46] software tool was used to perform the MD simulation and it is installed at the Computational Shared Facility (CSF3), University of Manchester, UK. Prior to simulation, each system was solvated using TIP3P [47] water model and immersed in a truncated octahedron box. A sufficient number of Na^+^ and Cl^−^ were added to neutralize the system. The physiological pH was retained by maintaining the ionic strength of 0.15 M. The protein forcefield, ff14SB [48] was applied to generate the protein topology. The GAFF2 [49] force field was used to generate the ligand topology. The PMEMD.CUDA module [50] was considered to perform the simulation. Throughout the simulation, a constant temperature of 300 K was retained using the Langevin thermostat with a collision frequency of 2 ps-1, at 1 atm using a Monte Carlo barostat with volume exchange attempts every 100 fs. The integration step was kept with a 2 fs step. The hydrogens associated with covalent bonds were constraint using the SHAKE [51] algorithm. A cut-off of 8 Å was considered for the short-range nonbonded interaction, while the particle mesh Ewald method [52] was used for the long-range electrostatics. A total of 10 ns time span equilibration was performed consisting of rounds of NVT and NPT. On successful completion of the MD simulation, several parameters such as root-mean-square deviation (RMSD) of the TMPRSS2 backbone, root-mean-square fluctuation (RMSF) and radius of gyration (RoG) were explored using CPPTRAJ [53] over full trajectory, taking configuration every 2 ps.

### Binding free energy calculation through MM-GBSA approach

There are two most trustworthy and widely used binding free energy calculations from MD simulation trajectory, namely Molecular Mechanics/Poisson-Boltzmann Surface Area (MM/PBSA) and the Molecular Mechanics/Generalized Born Surface Area (MM/GBSA) [54]. Both models calculate binding free energies by combining molecular mechanics calculations and continuum solvation models. Both approaches are used in a number of interesting studies [55–58] included by our research group [59–61]. Hou et al. [62] considered both the approaches and calculated binding free energy of several ligands. It was concluded that MM/GBSA gives better correlations than MM/PBSA in most systems. Hence, the MM-GBSA was used to calculate the binding free energy (*∆G*_bind_) of the TMPRSS2 inhibitors. The post-processed ensemble of structures are considered from the MD simulation trajectories to calculate the *∆G*_bind_ of each molecule. The following step by step expressions are used for *∆G*_bind_ calculation.

1 $$\Delta {G}_{\mathrm{bind}}={G}_{\mathrm{com}}-({G}_{\mathrm{rec}}+{G}_{\mathrm{lig}})$$

2 $${\Delta G}_{\mathrm{bind}}= \Delta H-T\Delta S$$

3 $${\Delta G}_{\mathrm{bind}}= {\Delta E}_{MM}+ {\Delta G}_{\mathrm{sol}}-T\Delta S$$

4 $${\Delta E}_{MM}= {\Delta E}_{\mathrm{int}}+ {\Delta E}_{\mathrm{ele}}+ {\Delta E}_{vdw}$$

5 $${\Delta G}_{\mathrm{sol}}= {\Delta G}_{\mathrm{pol}}+ {\Delta G}_{\mathrm{npol}}$$

Equation (1) is used to calculate the total binding energy (*∆G*_bind_). Basically, *∆G*_bind_ is the difference of free energy between complex (*∆G*_com_) and addition of the receptor (*∆G*_rec_) and ligand (*∆G*_lig_). The enthalpy (∆*H*) and entropy (*T*∆*S*) terms are associated in the *∆G*_bind_ (Eq. 2). The GBSA approach is used to get the enthalpy term. The normal mode analysis (NAM) and interaction entropy (IE) methods are considered to calculate the entropy. The ∆*H* is the term in which molecular mechanical energy (∆*E*_MM_) and solvation free energy (∆*E*_sol_) are involved. A combination of intra-molecular (∆*E*_int_), electrostatic (∆*E*_ele_) and the van der Waals interaction (∆*E*_vdw_) energies gives the ∆*E*_MM_. The addition of polar (∆*G*_pol_) and non-polar (∆*G*_npol_) energies gives the solvation (∆*G*_sol_). Both ∆*G*_pol_ and ∆*G*_npol_ obtained from the LCPO algorithm [63] which is based on SASA are calculated using the modified generalized Born (GB) [64] method.

## Results and discussion

### Virtual screening

**Fig. 1:**
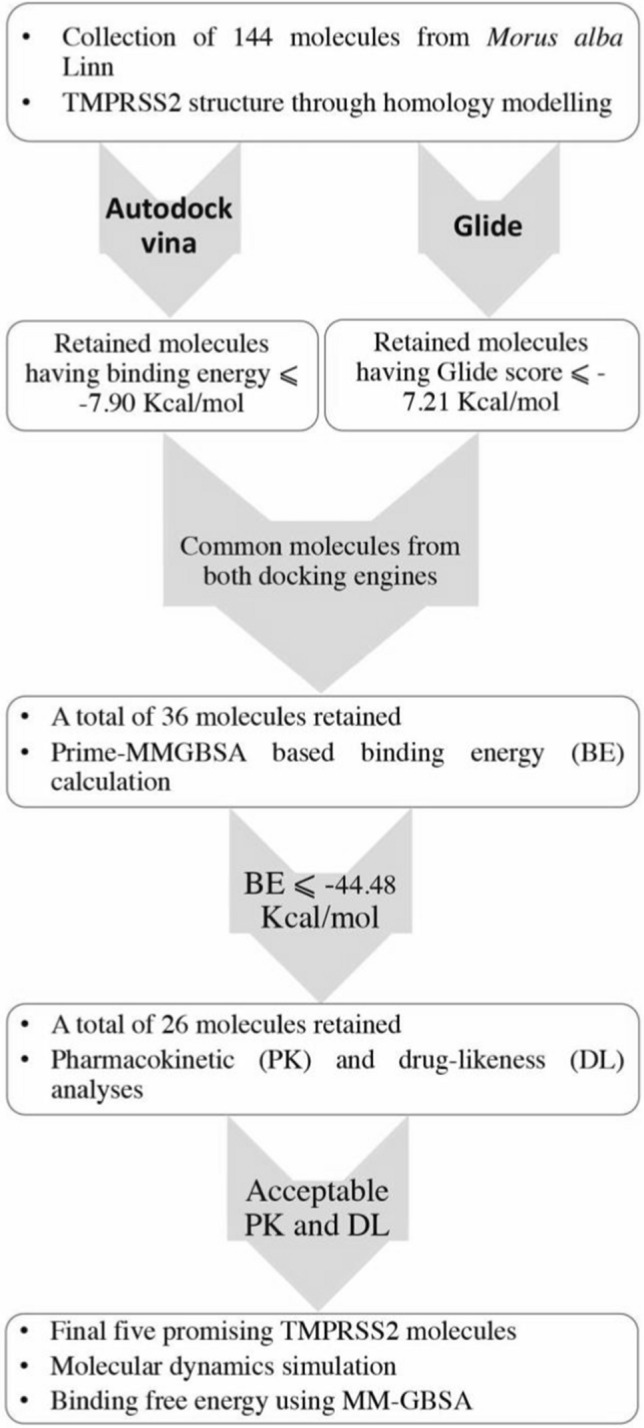
Virtual screening work of TMPRSS2 inhibitors

A set of 144 bioactive molecules isolated from the *M. alba* were used for screening against the TMPRSS2. A schematic flow diagram of the virtual screening is given in Fig. 1. The Glide score of two control molecules, Camostat and Ambroxol was found to be − 7.21 and − 6.23 kcal/mol, respectively. On docking of both Camostat and Ambroxol through ADV, the binding energy was obtained as − 7.90 and − 7.20 kcal/mol, respectively. Moreover, Prime-MMGBSA binding energy was found to be − 25.00 and − 44.48 kcal/mol for Camostat and Ambroxol, respectively. Hence, to reduce the chemical space, the threshold glide score, ADV binding energy and Prime-MMGBSA binding free energy were considered to be − 7.21, − 7.90 and − 44.48 kcal/mol, respectively.

**Fig. 2:**
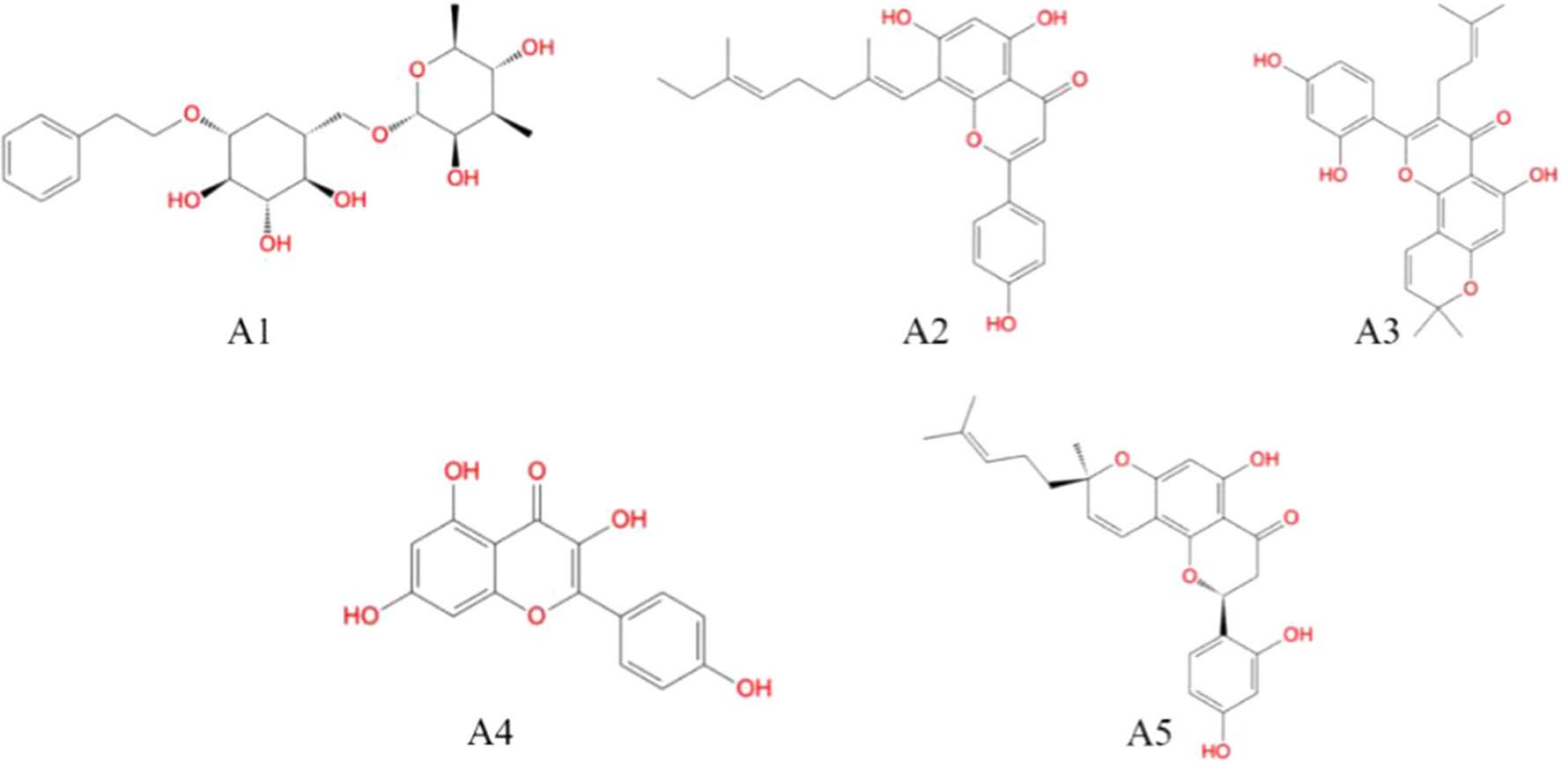
Two-dimensional representation of the selected bioactives compounds, A1, A2, A3, A4 and A5 from the *M. alba* considered potential anti-TMPRSS2 activity

Parallelly two molecular docking engines such as Glide and ADV were used to dock the entire set of small molecules in the TMPRSS2. On successful docking of the entire molecular set in TMPRSS2 through the Glide, molecules having glide score less than − 7.21 kcal/mol were considered. On the other hand, the binding energy of all molecules was explored after docking through ADV. Molecules found with higher binding energy than − 7.90 kcal/mol was removed and remaining carried forward for the next level of assessment. A total of 36 molecules were found common in both steps of Glide and ADV docking study and considered for further screening approaches. Moreover, the Prime-MMGBSA approach was used to calculate the binding free energy of each molecule. Molecules having higher Prime-MMGBSA-based binding free energy than − 44.48 kcal/mol were removed and a total of 26 molecules retained. The drug-likeness and pharmacokinetic parameters were assessed. Molecules violating LoF and Veber’s rules, having GI absorption = low or moderate, TPSA > 140 Å^2^, BBB permeation = yes and SA > 6 were removed. Following the above set of screening criteria, a total of five molecules such as (phenylethyl-D-rutinoside, 8-geranylapigenin, Morusin, Kaempferol and Sanggenol L) remained and considered to be promising TMPRSS2 inhibitors. For simplicity, from here onwards, phenylethyl-D-rutinoside, 8-geranylapigenin, Morusin, Kaempferol and Sanggenol L can be known as A1, A2, A3, A4 and A5, respectively. A two-dimensional representation of the final selected molecules is given in Fig. 2.

### Binding interactions analysis

**Fig. 3:**
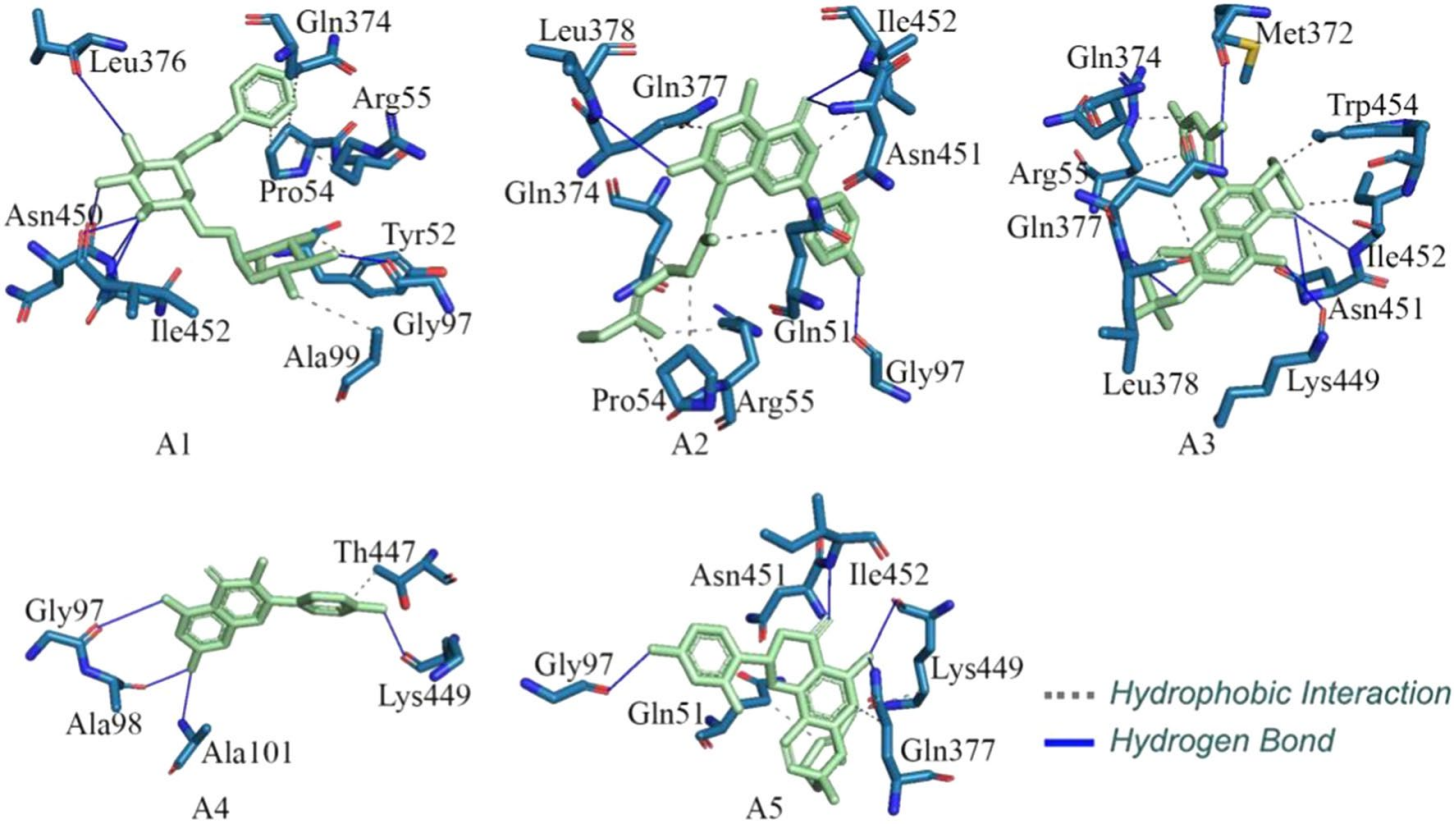
The binding interaction of A1, A2, A3, A4 and A5 with the active site of the TMPRSS2

**Fig. 4:**
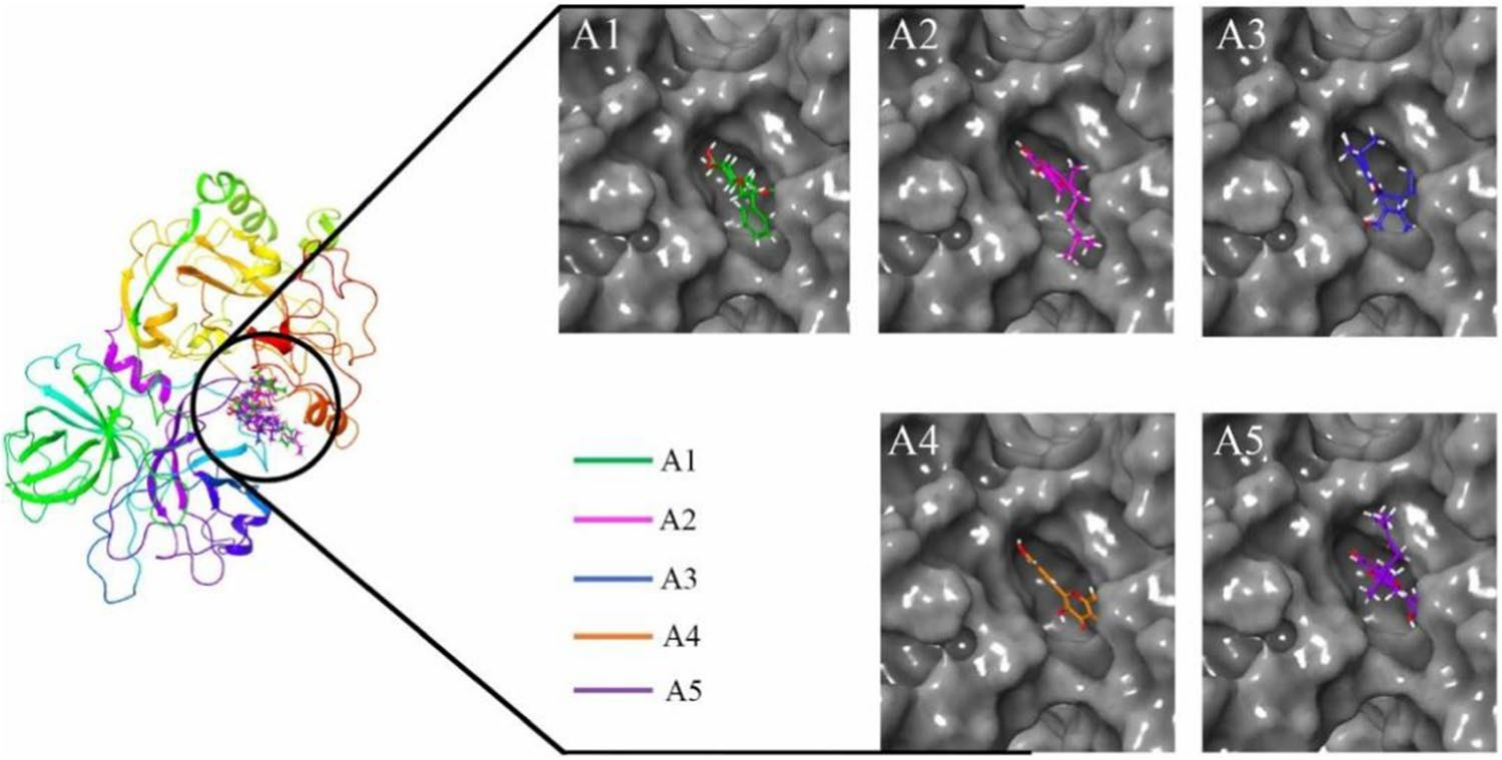
The binding mode of A1, A2, A3, A4 and A5 with the active site of the TMPRSS2

The glide XP score, ADV binding energy and Prime-MMGBSA binding energy of final molecules are given in Table 1. Several potential hydrogen bonds (HB) and non-bonding interactions were observed between the ligands (phytochemicals) and binding site amino residues of the TMPRSS2 shown in A1 of Figs. 3 and 4. Compound A1, phytochemical obtained from ethanolic extract of the *M. alba* fruits. On docking of A1 into the active site of TMPRSS2, its aliphatic side chain contains phenyl group hydrophobic interact with Pro54, Gln374 and Arg55 with a distance of 3.56 and 3.80 Å, respectively. Another pyran ring with two hydroxy and two methyl groups was interacting with Tyr52 and Ala99 through 1,1 HB interaction along with interatomic distance 3.84–3.99 Å. Furthermore, cyclohexane ring substituted with 3′ OH group was found critical to form one HB bond with Leu378 with the bond distance of 3.38 Å. In addition, cyclohexane ring substituted with 4′ OH group was also found crucial to interact with Asn450 and Asn451 through HB interactions.

**Table 1:** Dock score from Glide and Autodock vina, and binding energy from Prime-MMGBSA

Compound	Prime-MMGBSA BE	Glide dock score	Vina dock score
A1	− 70.472	− 9.574	− 8.80
A2	− 56.060	− 7.980	− 9.00
A3	− 50.510	− 7.888	− 8.90
A4	− 46.680	− 7.807	− 8.10
A5	− 60.830	− 7.426	− 9.30
Ambroxol	− 25.000	− 6.230	− 7.20
Camostat	− 44.480	− 7.210	− 7.90

Cyclohexane ring substituted with 5′ OH group was found to interact with Ile452 to form two HBs with a distance of 2.09 and 3.04 Å. Another pyran ring contains two hydroxyl and two methoxy functional groups was seen to form HB along with a distance of 2.44 Å. Furthermore, A1 interacted with the TMPRSS2 to with better affinity as suggested by the highest Prime-MMGBSA binding energy (− 70.472 kcal/mol), along with a Glide dock score (− 9.574 kcal/mol) and the ADV binding energy (− 8.80 kcal/mol). Compound A2, phytochemical investigation of the MeOH extract of the *M. alba* leaves and known as flavonoids. After successful docking of A2 into the active site of the TMPRSS2 (A2 in Figs. 3 and 4), its aliphatic side chain (2,6-dimethylocta-1,5-diene) interacted with Pro54, Arg55 and Gln51 with a distance of 3.44 and 3.61 Å, respectively. Another flavonoids ring with two hydroxyl groups and one ketone group were interacted with Ile452 and Asn451 with a distance of 3.92 and 2.83 Å, respectively. Moreover, flavonoids ring substituted with phenyl OH group was found to be important to interact with Gly97 via HB interactions. The flavonoids ring with two hydroxyl groups form three HBs with Leu378, Ile452 and Asn451. This compound showed better interaction with the TMPRSS2 as demonstrated by the calculated Prime-MMGBSA BE (− 56.060Kcal/mol), with an ADV binding energy (− 9.00 kcal/mol) and the Glide dock score (− 7.980 kcal/mol). Compound A3 is an extended flavonoid obtain from leaves *M. alba* and known as flavone substituted by hydroxy groups at positions 5, 2′ and 4′, a phenyl group at position 3 and a 2,2-dimethyl pyran group across positions 7 and 8. Docking results (A3 in Figs. 3 and 4) indicated that A3 contains an aliphatic side chain (2-methylbut-2-ene) substituted at a flavone ring to form HB with Trp454 with an interatomic distance of 3.57 Å. The ketonic functional group present in the flavone ring in Morusin was found to form HB interaction with Ile452 and Asn451 with a distance of 3.54–3.51 Å, respectively. In addition, the flavone 5 hydroxy group to form HB interactions with Arg55 and Gln374 with an interatomic distance of 3.49–3.63 Å. Another pyrin ring contains a methyl group present in the flavone ring to form HB interactions with Gln371 and Leu378 with interatomic distance 3.65–3.339 Å, respectively. Hydrogen bond interaction with residue Met372 was found with a flavone 2′ hydroxy group with a distance of 1.93 Å. Furthermore, the binding site of amino residues such as Ile452 and Asn451 were found to interact with the ketonic group of flavone through two hydrogen bonding with distance of 2.227 and 3.43 Å, respectively. Another pyrin ring contains carbon-hydrogen groups that interacted with Gln377 with a distance of 2.94 Å. In addition, the methyl group of pyrin ring was found to form one HB with Leu378 with a distance of 3.09 Å. The 5-hydroxyl functional group present in flavone was found to one HB with Lys449 with an interatomic distance of 2.05 Å. Furthermore, compound A3 interacted with the TMPRSS2 as shown by the calculated Prime-MMGBSA BE (− 50.510 kcal/mol), which was less than compound A1 and A2 but higher than compound A4 along with ADV binding energy (− 8.90 kcal/mol) and the Glide dock score (− 7.888 kcal/mol).

Compound A4 is a natural flavonoid that has been isolated from leaves *M. alba* and it is known as 3,4′,5,7-tetrahydroxyflavone. From the molecular docking study of A4, it was clearly indicated that A4 contains a phenyl ring substituted at flavone to form one HB interaction with Thr447 along with an interatomic distance of 3.90 Å. The 5-hydroxyl functional group present chromone ring in Kaempferol was found to form one HB with amino residues such as Gly97 with a distance of 2.75 Å. In addition, the binding site of amino residues such as Ala98 and Ala101 were found to interact with hydroxy groups present chromone ring through two HBs with the distance of 1.83 and 2.77 Å, respectively. One of the hydroxyl groups of phenyl ring linkage with chromone ring was formed one HB with Lys449 a distance of 2.03 Å. The Prime-MMGBSA binding energy of A4 was analyzed with their corresponding dock conformation at receptor binding sites. Results indicating that the Prime-MMGBSA binding energy of A4 (− 46.680 kcal/mol) was lesser than the other four compounds. Glide dock score (− 7.807 kcal/mol) lesser than the ADV binding energy (− 8.10 kcal/mol). Compound A5 is a natural flavonoid present in the root barks of *M. alba*. From the molecular docking of A5 into the active site of the receptor (A5 of Figs. 3 and 4), it was found that an aliphatic chain (4-methylpent-3-en-1-yl) form HB interacted with Gln51 and Lys449 with a distance of 3.37 and 3.90 Å, respectively. Furthermore, the carbon atoms of the chromone ring in the molecular system potentially was established the HB interaction with Gln377 with a distance of 3.94 Å. The ketonic functional group present in the chromone ring in A5 was found to form two HBs with amino residues such as Ile452 and Asn451 with an interatomic distance of 2.01 and 3.02 Å respectively. The 5 hydroxyl functional groups present in the chromone ring in Sanggenol L were found to form two H bonds with amino residues such as Lys449 and Gln377, respectively. Furthermore, Gly97 was found to interact with 4′ hydroxyphenyl group linkage with chromone ring through one HB interaction with a distance of 1.86. The binding energy of title phytochemical was analyzed by their docking conformation at the active binding site of the receptor. The results showed that Prime-MMGBSA binding energy of A5 (− 60.830 kcal/mol) was lower than compound A1 but higher than the compounds A2, A3 and A4. The glide dock score (− 7.426 kcal/mol) lower than the other four compounds and the ADV binding energy the (− 9.30 kcal/mol) higher than the other four phytochemicals. Thus, compound A1, A2, A3, A4 and A5 showed the highest Prime-MMGBSA binding energy and dock score as compared to both standard drugs (Camostat and Ambroxol) [17]. It is worth to note that no binding interaction was seen to form with catalytic triads in the molecular docking study. This might be due to unfavorable structural orientations of the proposed molecules at the active site cavity. It also can be observed that a number of nearby amino acids of catalytic triads were present in close proximity of the proposed ligands. The close proximate amino acids of the proposed TMPRSS2 modulators are given in Figure S1 (Supplementary data). Hence, optimization and/or different conformation analyses can form potential interactions with the catalytic triads.

### Pharmacokinetic and drug-likeness

The drug-likeness and pharmacokinetic parameters of the final five molecules are given in Table 2. It can be seen that all molecules follow the LoF and Veber’s rule. The TPSA was found to be in the range of 90–128 Å^2^ which is acceptable being a lead-like molecule. All molecules were found to be high penetrable to the gastro intestine. Not a single molecule was found to have synthetic accessibility of more than 6 which undoubtedly explained that all molecules can be synthesized easily.

**Table 2:** Pharmacokinetic and drug-likeness properties

Compound	Violation of LoF	GI absorption	TPSA	BBB permeation	Veber’s rule violation	Synthetic accessibility
A1	0	High	128.84	No	0	5.63
A2	0	High	90.90	No	0	4.24
A3	0	High	100.13	No	0	4.43
A4	0	High	111.13	No	0	3.14
A5	0	High	96.22	No	0	4.77

### Molecular dynamics simulation

The MD simulation becomes a pivotal and essential tool to explore a number of biological characteristics and dynamic behavior of the protein–ligand complex. The complex of small molecules bound protein systems are extremely important in biochemistry. Hence, the stability of the complex and the binding nature of the small molecules need to be explored through biochemical and biophysical approaches. For this purpose, all complexes of proposed molecules with TRMPSS2 were considered for subjected to 100 ns all-atoms MD simulation study in an explicit hydration environment. On successful completion, the MD simulation, the entire trajectory of each complex was analyzed in terms of a number of parameters included RMSD of protein backbone and ligand, RMSF of individual amino acid, hydrogen bond analysis between protein and ligand, RoG of the system and finally, the binding affinity of the molecules in terms of binding energy calculated through MM-GBSA approach.

#### Root mean-square deviation

**Fig. 5:**
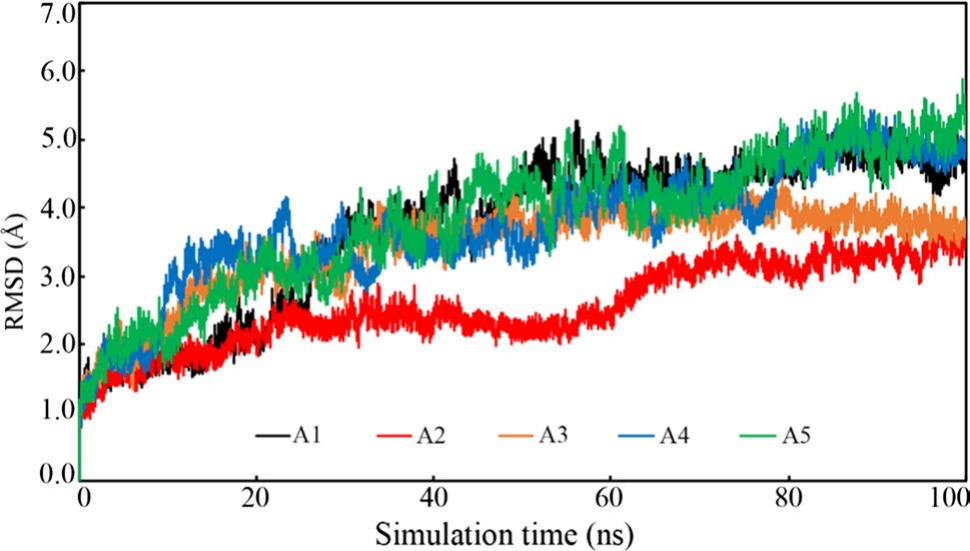
TMPRSS2 backbone RMSD bound with proposed molecules

The structural conformation of the protein backbone during the MD simulation can be assessed through RMDS of each frame obtained from the entire trajectory. RMSD of Ca, C and N atoms each frame was calculated and plotted against the simulation time and it is given in Fig. 5. The stability protein–ligand complex can be explained by the low deviation and constant variation of the RMSD throughout the simulation. From Fig. 5, it can be seen that initially the RMSD deviated but afterward all complexes achieved stability. It is important to note that all complexes were found to a gradual increase of RMSD un till about 20 ns of simulation time. Followed by about 20 ns all complexes were seen to equilibrated with small deviation which indicated that systems folded in more stable conditions in comparison to the native structure. Average, maximum and minimum RMSD was calculated and these are given in Table 3. The average TMPRSS2 backbone RMSD was found to be 3.702, 2.550. 3.421, 3.730 and 3.8832 Å when bound with A1, A2, A3, A4 and A5, respectively. The same protein bound with Camostat and Ambroxol was given an average RMSD of 3.265 and 3.909 Å, respectively [17]. The above data clearly suggest that the average RMSD of TMPRSS2 backbone bound with proposed molecules given almost similar deviation to the control molecules.

**Table 3:** Average, maximum and minimum values of MD simulation parameters

Parameters	A1	A2	A3	A4	A5
*RMSD (Å)*					
TMPRSS2 (Ca, C and N)					
Average	3.702	2.550	3.421	3.730	3.832
Maximum	5.286	3.811	4.446	5.437	5.887
Minimum	0.000	0.000	0.000	0.000	0.000
Ligand					
Average	1.634	0.378	0.311	0.289	0.313
Maximum	2.734	1.375	1.238	1.310	0.688
Minimum	0.000	0.000	0.000	0.000	0.000
*RMSF (Å)*					
Average	1.898	1.595	1.809	1.719	2.001
Maximum	13.293	9.379	9.486	8.344	12.260
Minimum	0.541	0.488	0.535	0.597	0.737
*RoG (Å)*					
Average	24.280	23.965	24.624	23.791	24.677
Maximum	24.942	24.568	25.229	24.315	25.308
Minimum	23.779	23.525	23.915	23.373	24.005

**Table 4:** Binding free energy of proposed TMPRSS2 inhibitors

Molecule	Energy (Kcal/mol)			Standard error of *∆G*_*bind*_
	^a^Elec	^b^vdW	*∆G*_*bind*_	
A1	− 37.317	− 43.564	− 36.546	4.726
A2	− 19.191	− 51.521	− 42.252	3.370
A3	− 18.494	− 45.633	− 36.534	2.617
A4	− 25.883	− 35.702	− 30.462	3.079
A5	− 25.566	− 50.009	− 42.916	3.845

^a^Electrostatic

^b^ven der Waal’s

#### Root-mean square fluctuation

**Fig. 6:**
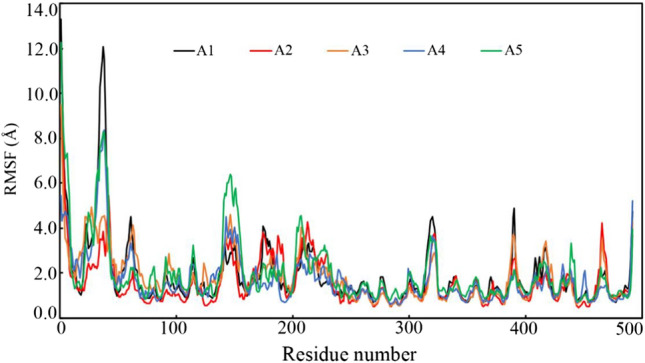
RMSF of individual amino residue of TMPRSS2 bound with A1, A2, A3, A4 and A5

The RMSF parameter is extremely essential to explore the role of individual amino acid in the stability of any protein–ligand complex. It is the fluctuation of each amino acid backbone during the simulation with respect to the initial orientation in the native state. The RMSF of each amino acid was calculated from the MD simulation trajectory and it is given in Fig. 6. The average, maximum and minimum RMSF values are given in Table 3. It can be seen that all the trajectories fluctuated almost in a similar fashion with a small variation. Amino acid around 25 of TMPRSS2 bound with A1 and A5 were found to fluctuated higher in comparison to others but the remaining residues are shown similar fluctuation. It is important to note that similar fluctuation of TMPRSS2 was observed when it bounds to the Ambroxol and Camostat [17]. A little bit higher fluctuations were observed around Pro30, Arg150, Met320, Lys390 and Phe480 in comparison to the other amino acids. The above higher fluctuation might be due to the breaking of binding interactions between the proposed molecules and ligand-binding amino residues in the dynamic states during MD simulation. The average RMSF value can give an idea about the fluctuation of the amino residues during the simulation. From Table 3, it can be seen the average RMSF value of 1.898, 1.595, 1.809, 1.719 and 2.001 Å was observed when bound to A1, A2, A3, A4 and A5, respectively. The above RMSF data undoubtedly suggested that during the simulation, amino residues of TMPRSS2 bound with proposed molecules remained consistent.

#### Radius of gyration

**Fig. 7:**
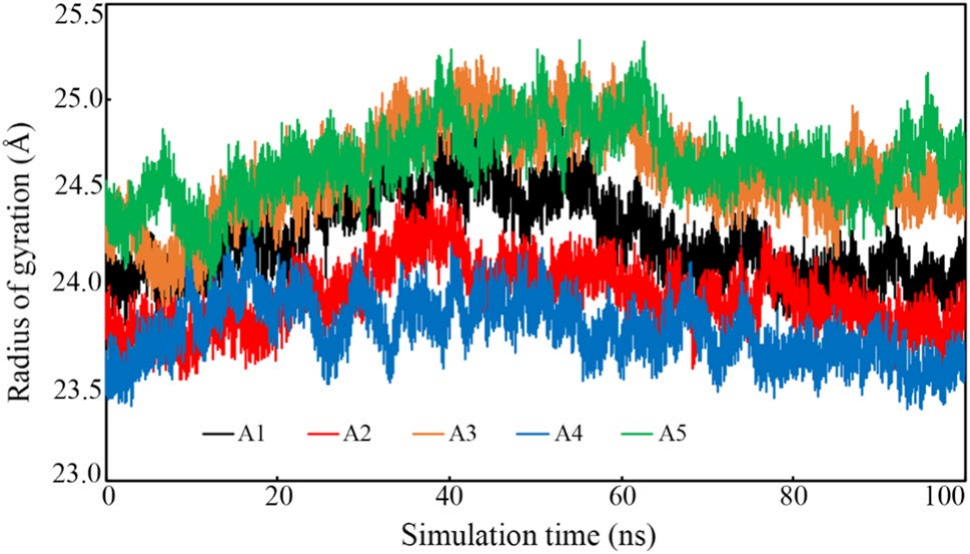
Radius of gyration against simulation time of TMPRSS2 bound with A1, A2, A3, A4 and A5

The rigidity comparative analysis of protein-bound with small molecules can be assessed through the radius of gyration. It is reported that almost intact RoG variation explain the folding of the protein during the MD simulation. On the contrary, the high deviation of RoG describes the unfolding of the macromolecules. The RoG of each frame of the TMPRSS2 complex with the final proposed molecules was extracted and plotted against the time of simulation (Fig. 7). The pattern of RoG variation for each system clearly indicated the consistency during the simulation. No abnormal variation of the RoG value was found in any of the systems. The difference between the maximum and minimum RoG was found to be 1.163, 1.043, 1.314, 0.942 and 1.303 Å when TMPRSS2 bound with A1, A2, A3, A4 and A5, respectively. Such a low difference undoubtedly explained that not abnormal opening of the protein was seen.

#### Hydrogen bond analysis

**Fig. 8:**
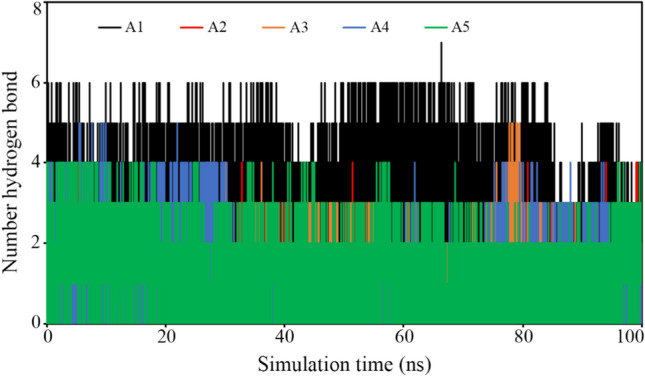
The number of HBs forming between TMPRSS2 and proposed ligands in due course of simulation time

The protein–ligand complex stability and affinity of the ligand toward the receptor can be assessed the hydrogen bond analysis of each frame generated during the MD simulation. The hydrogen bonds formed by each ligand with TMPRSS2 in each frame were calculated and it is given in Fig. 8. The maximum number of hydrogen bonds formed by A1, A2, A3, A4 and A5 with TMPRSS2 was found to be 7, 4, 5, 5 and 4, respectively. In each and every molecule, a small number of frames were also found without any hydrogen bonds but those frames were remained stabled with non-hydrogen bond interactions.

Idris et al. [65] were performed the pharmacophore-based virtual screening of the ZINC database against TMPRSS2 target. After molecular docking and *in-silico* pharmacokinetic analyses, they were found two promising molecules. A 50 ns time span of MD simulation was performed for the complex of TMPRSS2 and the final two molecules. Average protein backbone RMSD they have reported as 4.52 and 5.28 Å bound with ligand1 and ligand2, respectively. In the current study, the average TMPRSS2 backbone RMSD bound with all five molecules was found to be in the range of 2.5 to 3.9 Å. The Average RMSF was reported for ligand1 and ligand2 as of 1.87 and 2.06 Å, respectively. Similar to the above average RMSF (< 2.002 Å) was seen for the TMPRSS2 bound with A1–A5. In the above study, the average RoG of ligand1 and ligand2 was found to be 20.63 and 20.32 Å, respectively. Mean RoG of TMPRSS2 bound with proposed molecules (A1–A5) was seen to be less than 25 Å. The RMSD and RMSF profile of the TMPRSS2 inhibitors in our previous publication [17] were also corroborated with the current findings.

### Binding energy calculation using MM-GBSA approach

Binding free calculation through MM-GBSA from a set of frames obtained in MD simulation can be considered more accurate and trustworthy in comparison to the molecular docking study. Hence, for each proposed molecule, last 10,000 frames were used to calculate the Δ*G*_bind_ through MM-GBSA approach. The Δ*G*_bind_ along with different components of the binding free energy was calculated and these are given in Table 4. The same approach was used in our previous study [17] to calculate the Δ*G*_bind_ of Ambroxol and Camostat, and the value was found to be − 44.480 and − 25.00 kcal/mol respectively. In the current study, Δ*G*_bind_ of A1, A2, A3, A4 and A4 was found to be − 36.546, − 42.252, − 36.534, − 30.462 and 42.916 kcal/mol, respectively. It is quite interesting that Δ*G*_bind_ of all molecules was found to be more than the binding free energy of Camostat and comparable with Ambroxol. The above value clearly indicated that all proposed molecules were showed strong affection toward the TMPRSS2.

## Conclusion

In the current study, five promising molecules were obtained through structure-based virtual screening for TMPRSS2 inhibition. The potentiality of each molecule was adjudged through binding interaction, *in-silico* pharmacokinetic and MD simulation assessments. The molecular docking study was clearly explained that a number of crucial amino acids including Arg55, Glu374, Leu376, Asn451, etc. found to be critical to hold the ligands inside the active site of TMPRSS2. The pharmacokinetics and drug-likeness characteristics of each ligand suggested the potentiality of the selected molecules. To check the behavior of each molecule in the dynamic state, an all-atom MD simulation was performed. A number of parameters included RMSD, RMSF, RoG and hydrogen bond analysis were suggested the stability of protein–ligand complexes. MD simulation trajectory was used to calculate the binding free energy through the MM-GBSA approach. The high binding free energy of each molecule obtained from the above approach undoubtedly was explained the strong affection toward the TMPRSS2. Hence, the final proposed molecule might be crucial for TMPRSS2 inhibition and can be used for the management of COVID19, subjected to experimental validations.

## Supplementary Information

Below is the link to the electronic supplementary material.

Supplementary file1 (DOCX 2762 kb)

## Data Availability

Not applicable.
