# Supplementary material for: Pharmacoinformatics-based identification of transmembrane protease serine-2 inhibitors from Morus Alba as SARS-CoV-2 cell entry inhibitors
Source: Mol Divers. 2021 Mar 30;26(1):265–78. doi: 10.1007/s11030-021-10209-3 (PMC8009078; doi:10.1007/s11030-021-10209-3)
Supplement: Supplementary file 1 — Supplementary file1 (DOCX 2762 kb) [file 11030_2021_10209_MOESM1_ESM.docx]

**Pharmacoinformatics-based Identification of Transmembrane Protease Serine-2 Inhibitors from *Morus Alba* as SARS-CoV-2 cell entry Inhibitors**

Anshul Shakya, Rupesh V. Chikhale, Hans Raj Bhat, Fatmah Ali Asmary, Tahani Mazyad Almutairi, Surajit Kumar Ghosh^1^ Hassna Mohammed Alhajri, Siham A. Alissa, Md Ataul Islam

***Supplementary data***

**Table S1: Structures of phytoconstituents reported to be present in *M. alba* Linn.**

| **S. No.** | **Code** | **Structure** | **Name** |
| --- | --- | --- | --- |
|  | l1 | **** | Moralsin |
|  | l2 | **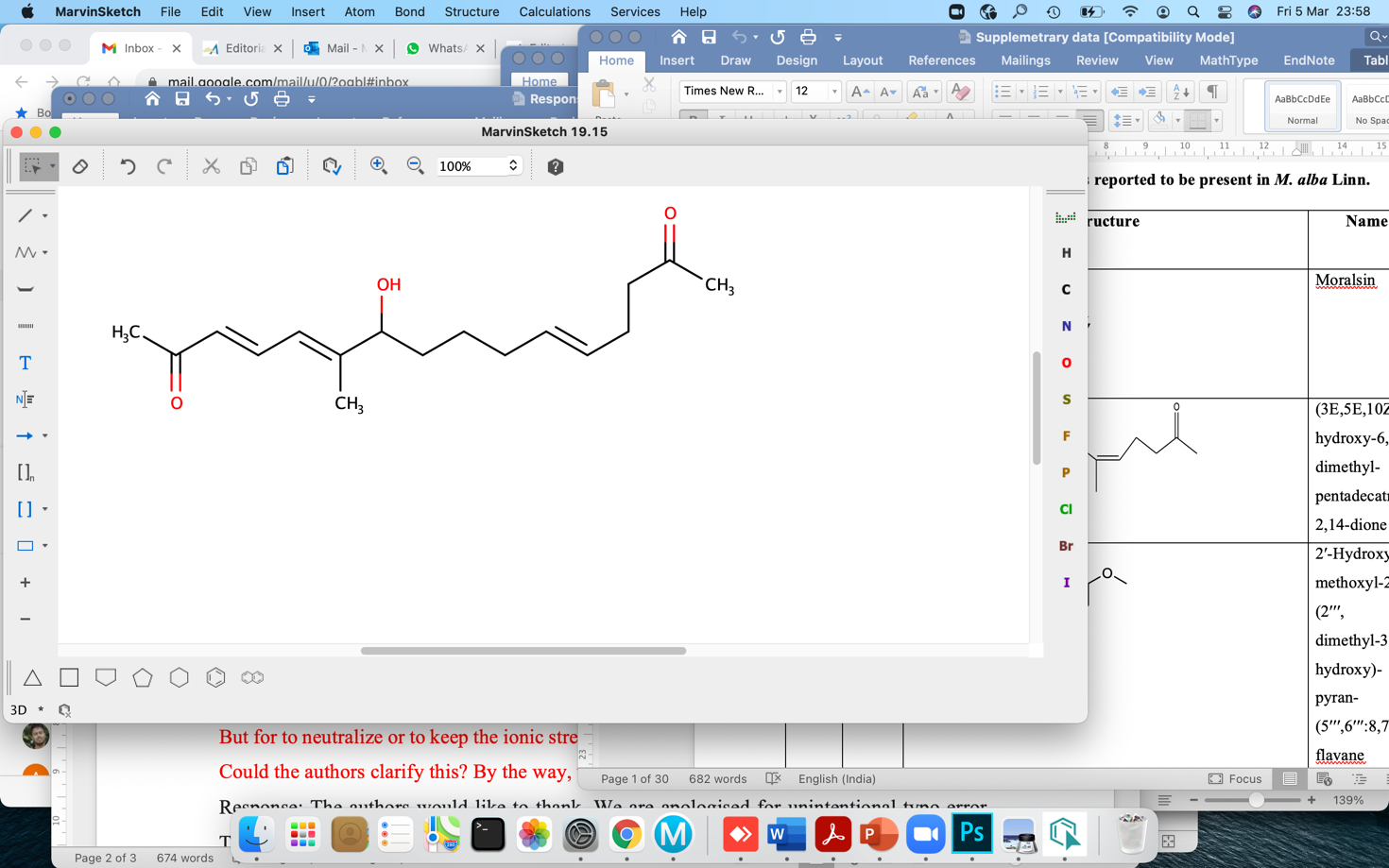** | (3E,5E,10Z)-7-hydroxy-6, 10-dimethyl-pentadecatrien-2,14-dione |
|  | l3 | **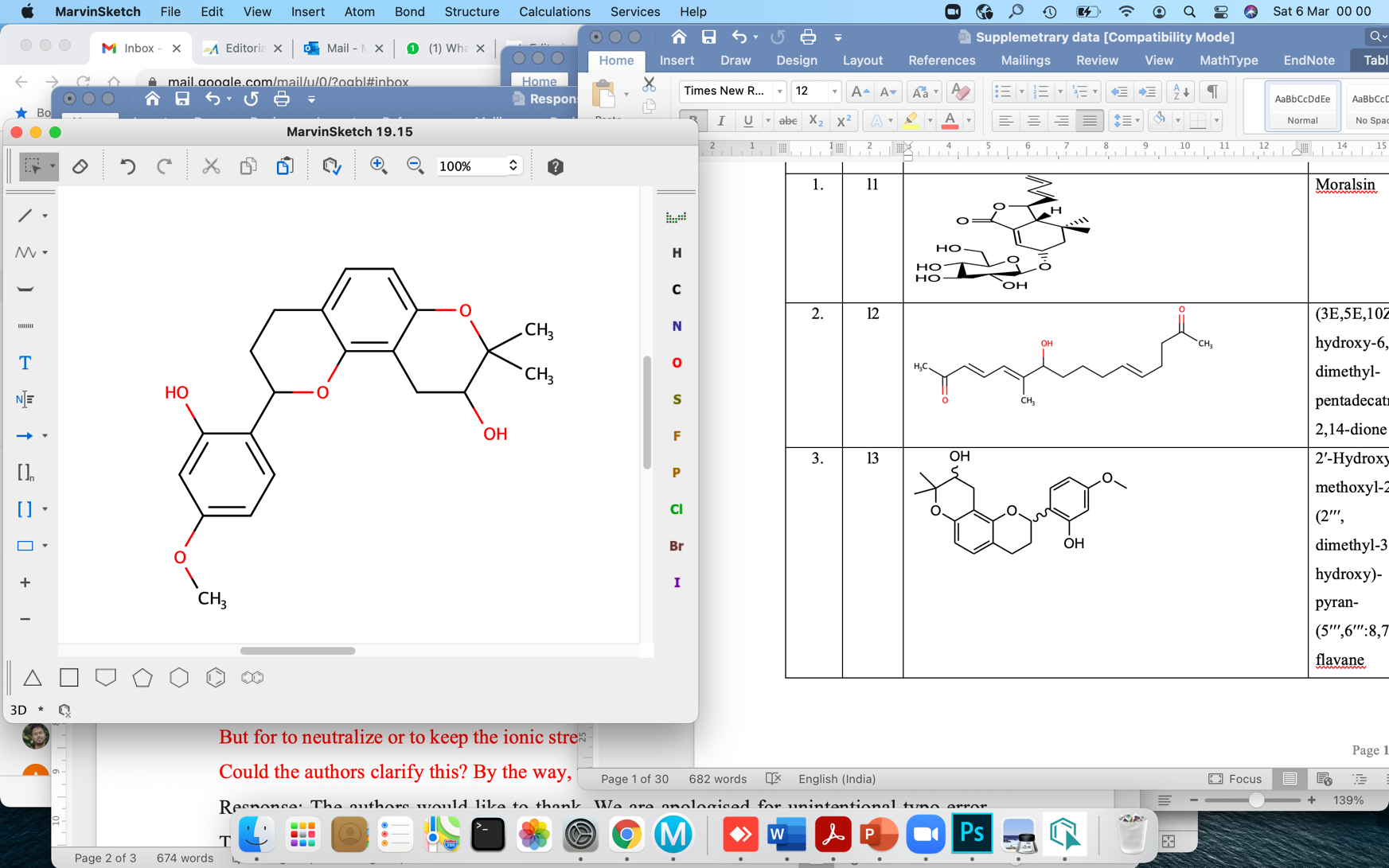** | 2′-Hydroxy-4′-methoxyl-2H-(2′′′, 2′′′-dimethyl-3′′′-hydroxy)- pyran-(5′′′,6′′′:8,7)-flavane |
|  | l4 | **** | Benzyl 2-O-[β-D-apiofuranosyl(1→6)-β-d-glucopyranosyl]-2,6-dihydroxybenzoate |
|  | l5 | **** | (9R)-Hydroxy-(10E,12Z,15Z)-octadecatrienoic acid |
|  | l6 | **** | 3-O-caffeoylquinic acid/ Chlorogenic acid |
|  | l7 | **** | 4-O-caffeoylquinic acid/ Cryptochlorogenic acid |
|  | l8 | **** | Gastrodin |
|  | l9 | **** | Quercetin 3-O-glucoside/Isoquercetin |
|  | l10 | **** | 1-Deoxynojirimycin |
|  | l11 | **** | 3′-geranyl-3-prenyl-2′,4′,5,7-tetrahydroxyflavone |
|  | l12 | **** | 3′,8-diprenyl-4′,5,7-trihydroxyflavone |
|  | l13 | **** | 8-geranylapigenin |
|  | l14 | **** | Cyclomulberrin |
|  | l15 | **** | Sanggenon J |
|  | l16 | **** | Sanggenon K |
|  | l17 | **** | Morusin |
|  | l18 | **** | Atalantoflavone |
|  | l19 | **** | Kaempferol |
|  | l20 | **** | Quercetin 3-O-rutinoside/ Rutin |
|  | l21 | **** | Kaempferol 3-O-D-glucoside/ Astragalin |
|  | l22 | **** | Quercetin |
|  | l23 | **** | Dihydroxybenzoic acid/ Protocatechuic acid |
|  | l24 | **** | Kuwanon S |
|  | l25 | **** | Moracin C |
|  | l26 |  | Caffeic acid |
|  | l27 |  | Malic acid |
|  | l28 |  | Citric acid |
|  | l29 |  | Ascorbic acid/ Vitamin C |
|  | l30 |  | Qunic acid |
|  | l31 |  | β- carotene |
|  | l32 | **** | Kaempferol 3-O-D-rutinoside/ Nicotiflorin |
|  | l33 | **** | Hesperidin |
|  | l34 |  | Morin |
|  | l35 |  | Quercetin 3,7- di-O- glucoside |
|  | l36 |  | Quercetin 3-O- rutinoside-7-O-glucoside/ Morkotin A |
|  | l37 |  | Quercetin 3-O- rutinoside- 7- O- rhamnoside/ Morkotin B |
|  | l38 |  | Kaempferol 3, 7, di- O- glucoside |
|  | l39 |  | Kaempferol 3-O-rutinoside 7-O-rhamnoside/ Moragrol B |
|  | l40 |  | 5- caffeoylquinic acid/ Neochlorogenic acid |
|  | l41 |  | Quercetin-di- hexoside |
|  | l42 |  | Kaempferol-di-hexoside |
|  | f1 |  | Butyl pyroglutamate |
|  | f2 |  | Kaempferol3-O-D-glucoside/Astragalin |
|  | f5 |  | 2-phenylethyl D-rutinoside |
|  | f6 |  | 4-[formyl-5 (methoxymethyl)-1H-pyrrol-1-yl]butanoate |
|  | f7 |  | 4-[formyl-5-(hydroxylmethyl)-1H-pyrrol-1-yl]butanoate |
|  | f8 |  | 4-[formyl-5-(methoxymethyl)-1H-pyrrol-1-yl]butanoic acid |
|  | f9 |  | [2-formyl-5-(hydroxyl methyl)-1 H pyrrol-1-yl] propanoic acid/ morrole B |
|  | f10 |  | 2[2-formyl-(methoxymethyl)-1 H pyrrol-1yl] propanoic acid/ morrole C |
|  | f11 |  | Methyl 2-[2-formyl-5-(methoxymethyl)-1H-pyrrole-1-yl]propanoate |
|  | f12 |  | 4-amino-2[2-formyl-5-(methoxymethyl)-1H-pyrrol-1yl)-4oxo butanoate/ morrole E |
|  | f13 |  | Methyl 2-[2-formyl-5-(methoxymethyl)-1H-pyrrol-1-yl]-3-(4-hydroxyphenyl)propanoate |
|  | f14 |  | 2-(5-hydroxymethyl-2-formylpyrrole-1-yl)propionic acid lactone |
|  | f15 |  | 2-(5-hydroxymethyl-2-formylpyrrol-1-yl)isovaleric acid lactone |
|  | f16 |  | 2-(5-hydroxymethyl-2-formylpyrrole-1-yl)isocaproic acid lactone |
|  | f17 |  | 2-(5’-hydroxymethyl-2’-formylpyrrol-1’-yl)-3-phenyl-propionic acid lactone |
|  | f18 |  | 2-[2-formyl-5-(hydroxymethyl)-1-pyrrolyl-]3-methylpentanoic acid lactone |
|  | f19 |  | 2-(5’-hydroxymethyl-2’-formylpyrrol-1’-yl)-3-(4-hydroxyphenyl)-propionic acid lactone |
|  | f20 |  | 2-(5-hydroxymethyl-20’,5’-dioxo-2’,3’,4’,5’-tetrahydro-1’H-1,3’-bipyrrole)carbaldehyde |
|  | f21 |  | Morrole G |
|  | f22 |  | 1-(2,6-dioxopiperidin -3-yl)-5-(hydroxylmethyl)-1H pyrrole-2-carbaldehyde/ morrole H |
|  | f23 |  | 5(hydroxymethyl)-1H-pyrrole-2-carboxaldehyde |
|  | f24 |  | 2-formyl-1H-pyrrole-1-butanoicacid |
|  | f25 |  | 2-formyl-5-(hydroxymethyl)-1H-pyrrole-1-butanoicacid |
|  | f26 |  | 2-formyl-5-(methoxymethyl)-1H-pyrrole-1-butanoicacid |
|  | f27 |  | MorroleA |
|  | f29 |  | Quercetin 3-O-(6ʺ-O-acetyl)-β-D-glucopyranoside |
|  | f30 |  | Quercetin 3-O-β-D-rutinoside |
|  | f31 |  | Quercetin 7-O-β-D-glucopyranoside |
|  | f32 |  | Quercetin 3,7-di-O-β-D-glucopyranoside |
|  | f33 |  | Kaempferol 3-O-β-D-glucopyranoside |
|  | f34 |  | 5,7,3´- trihydroxy - flavanone-4´-O-β-D-glucopyranoside |
|  | f35 |  | 5,7,4´- trihydroxy – flavanone - 3´ - O – β – D – glucopyranoside |
|  | f36 |  | Dihydrokaempferol 7-O-β-D-glucopyranoside |
|  | f37 |  | Isobavachalcone |
|  | f38 |  | 2,4,2´,4´,tetrahydroxy-3´-(3-methyl-2-butenyl) - chalcone / morachalcone |
|  | f39 |  | (2E)-1-[2, 3-dihydro-4-hydroxy-2-(1-methylethenyl)-5-benzofuranyl]-3-(4-hydroxyphenyl) - 1-propanone |
|  | f40 |  | 2-O-(3,4-dihydroxybenzoyl)-2,4,6-trihydroxyphenylacetic acid |
|  | f41 |  | 2-O-(3,4-dihydroxybenzoyl)-2,4,6-  trihydroxy phenyl methyl acetate/ jaboticabin |
|  | f42 |  | p-hydroxybenzoic  acid |
|  | f44 |  | 3-methoxy-4-hydroxybenzoic acid/ vanillic acid |
|  | f45 |  | Protocatechuic acid methyl ester |
|  | f46 |  | Protocatechuic acid ethyl ester |
|  | f47 |  | 4-hydroxyphenylacetic acid methyl ester |
|  | f48 |  | 5,7-dihydroxychromone |
|  | f49 |  | 2-(4-hydroxyphenyl)ethanol/ tyrosol |
|  | f57 |  | Leuteolin |
|  | f58 |  | Pyrocatechol |
|  | f60 |  | Cholecalciferol/ Vitamin D |
|  | f61 |  | Glutathione |
|  | r1 | **** | Kuwanon G |
|  | r2 | **** | Mulberrofuran G |
|  | r3 | **** | Albanol B |
|  | r4 | **** | Moralbanone |
|  | r6 | **** | Mulberroside C |
|  | r7 | **** | Cyclomorusin |
|  | r8 | **** | Eudraflavone B hydroperoxide |
|  | r9 | **** | Oxydihydromorusin |
|  | r10 | **** | Leachianone G |
|  | r11 | **** | α-acetyl-amyrin |
|  | r12 | **** | Morusalfuran A |
|  | r13 |  | Morusalfuran B |
|  | r14 |  | Morusalfuran C |
|  | r15 |  | Morusalfuran D |
|  | r16 |  | Morusalfuran E |
|  | r17 |  | Morusalfuran F |
|  | r18 |  | Morusalfuran G |
|  | r19 |  | Morusalnol A |
|  | r20 |  | Morusalnol B |
|  | r21 |  | Morusalnol C |
|  | r22 |  | Morusibene A |
|  | r23 |  | Morescoumarin A |
|  | r24 |  | Morflavanone A |
|  | r25 |  | Sanggenol Q |
|  | r26 |  | Sanggenol A |
|  | r27 |  | Sanggenol L |
|  | r28 |  | Kuwanon T |
|  | r29 |  | Sanggenon F |
|  | r30 |  | Sanggenol O |
|  | r31 |  | Sanggenon N |
|  | r32 |  | Sanggenon G |
|  | r33 |  | Mulberrofuran C |
|  | r34 |  | Moracin E |
|  | r35 |  | Betulinic acid |
|  | r37 |  | Moracin M |
|  | r39 |  | Mulberrofuran D |
|  | r40 |  | Albanol A |
|  | r41 |  | Mulberroside A |
|  | r43 |  | Kuwanon C |
|  | b1 |  | Linoleiyldiglycoside |
|  | b2 |  | Morusflavonyl palmitate |
|  | b3 |  | Morusflavone |
|  | b4 |  | Albosteroid |
|  | b8 |  | Apigenin |
|  | b9 |  | Oxyresveratrol |
|  | b10 |  | Dihydroxyresveratrol |
|  | b13 |  | Resorcinol |
|  | b14 |  | trans- dihydromorin |
|  | b15 |  | 2, 4- dihydroxybenzaldehyde |
|  | b16 |  | 2,34,3’- trihydroxydihydrostilbene |
|  | b17 |  | Kuwanon H |
|  | b20 |  | Cyclobutanol |
|  | b21 |  | Xanthatin |

**
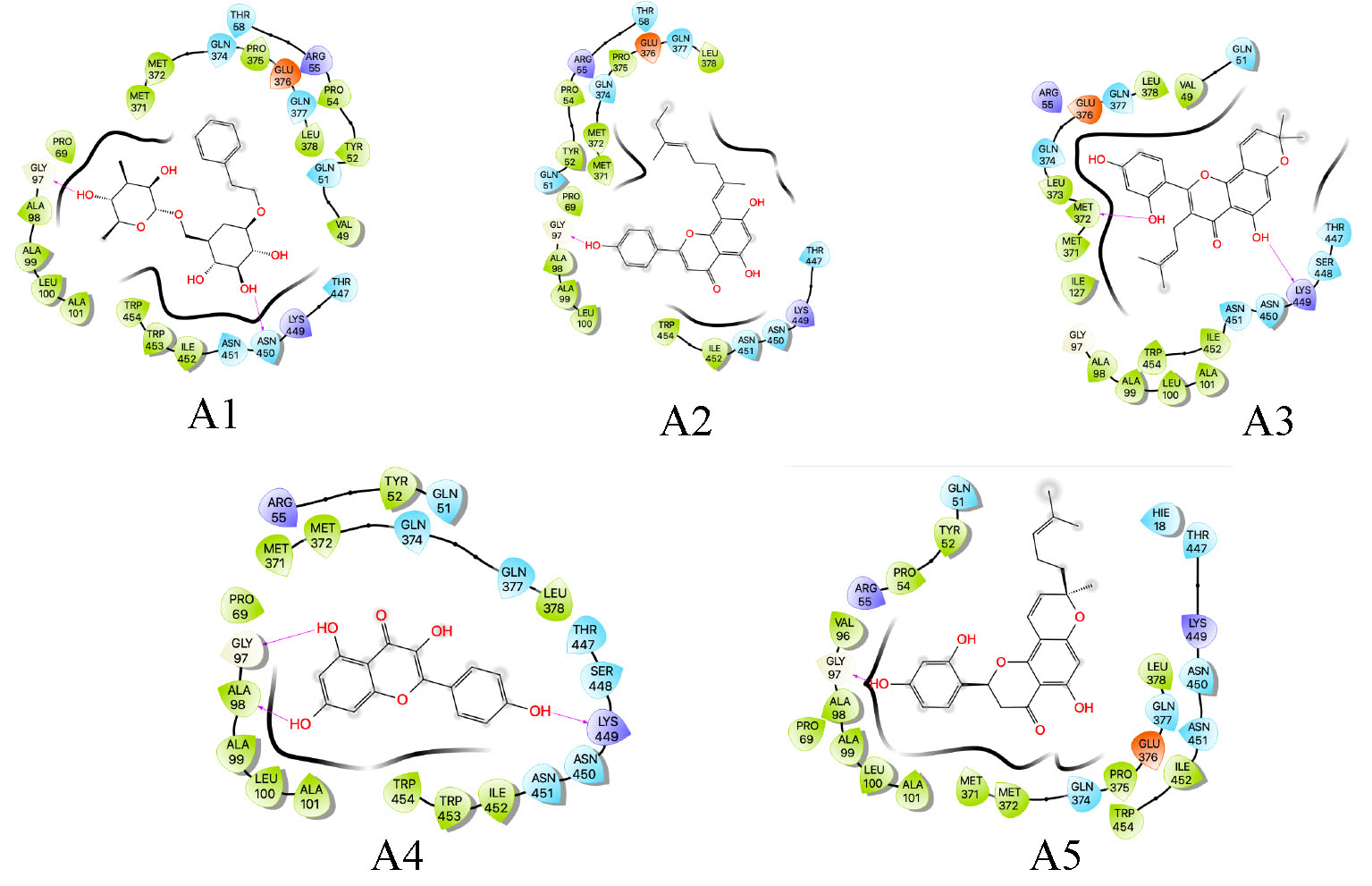
**

**Figure S1.** Presence of amino acids in close proximity of the proposed TMPRSS2 modulators
